# FDM Layering Deposition Effects on Mechanical Response of TPU Lattice Structures

**DOI:** 10.3390/ma14195645

**Published:** 2021-09-28

**Authors:** Chiara Ursini, Luca Collini

**Affiliations:** Department of Engineering and Architecture, University of Parma, Viale delle Scienze 181/A, 43124 Parma, Italy; luca.collini@unipr.it

**Keywords:** additive manufacturing, fused deposition modeling, lattice structures, TPU, layering

## Abstract

Nowadays, fused deposition modeling additive technology is becoming more and more popular in parts manufacturing due to its ability to reproduce complex geometries with many different thermoplastic materials, such as the TPU. On the other hand, objects obtained through this technology are mainly used for prototyping activities. For this reason, analyzing the functional behavior of FDM parts is still a topic of great interest. Many studies are conducted to broaden the spectrum of materials used to ensure an ever-increasing use of FDM in various production scenarios. In this study, the effects of several phenomena that influence the mechanical properties of printed lattice structures additively obtained by FDM are evaluated. Three different configurations of lattice structures with designs developed from unit cells were analyzed both experimentally and numerically. As the main result of the study, several parameters of the FDM process and their correlation were identified as possible detrimental factors of the mechanical properties by about 50% of the same parts used as isotropic cell solids. The best parameter configurations in terms of mechanical response were then highlighted by numerical analysis.

## 1. Introduction

As opposed to traditional production, which is mainly subtractive in nature, additive manufacturing represents the idea of future production, thanks to its considerable savings in materials and the extended possibilities in producing complex geometries. The major advantage of the fused deposition modeling (FDM) additive process lies in the customized production of printed objects. At the same time, various limitations prevent their dominance in the production of fully functional mechanical components, for example, the limited size of produced parts, but the cost should not be underestimated as well [1]. Furthermore, many printing process parameters have an influence on the resulting microstructure of printed objects, as observed by Ziemian et al. and Durgun et al. and discussed below [2,3].

FDM additive process is based on the extrusion of material and on the principle of stacking layer by layer, in order to create parts that can have sophisticated 3D geometries. Due to this principle of deposition of the material, this technology presents some pitfalls. In fact, by building FDM parts from bottom to top, the material in the current layer solidifies before the next one is placed on it [4], causing incomplete interlayer adhesion and often leaving voids in solidified structure, resulting in a decrease in mechanical performance [5]. In addition to this, a combination of several parameters such as raster orientation, air gap, bead width, color, model temperature, infill, etc. causes a decrease in the compressive strength of FDM products [6,7]. The layering effect represents the main challenge in additive manufacturing with fused deposition technology and will be analyzed in depth in the discussion below.

**Figure 1:**
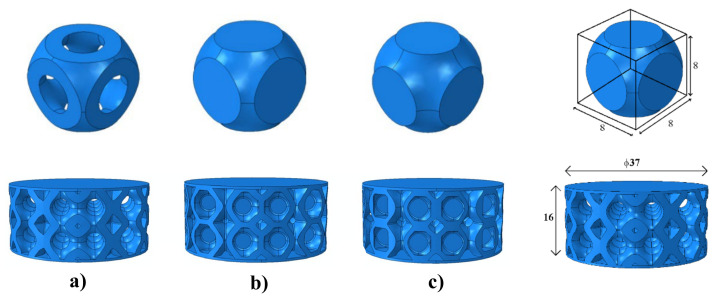
Unit cells and, respectively, lattice structures with their appropriate geometries and sizes [mm]: (**a**) open cell; (**b**) closed thin-walled cell; (**c**) closed thick-walled cell.

In this study, the printed parts under examination were lattice structures made of the repetition of three different unit cells with the same relative density—namely, open cell, closed thin-walled cell, and closed thick-walled cell. The unit cells repeated with the principle of tessellation along the three principal directions constitute the resulting lattice structures [8] (Figure 1). Fabrication of these structures by conventional manufacturing is not trivial, but Kumar et al. have found that with additive technologies, it is possible to produce them without any support structure [9]. An undoubted advantage of lattice structures, which are basically metamaterials since they are specifically designed to impart special physical properties normally absent in the constituent materials [10], is lightness, often a valuable attribute in engineering applications [11]. The material here used for printing is the thermoplastic polyurethane (TPU) 90A, which has hyperelastic and viscoelastic properties [12]. Three specimens of each configuration were additively realized and were tested under compression loads. Comparative analysis among the three configurations was carried out based on the mechanical property of stiffness. At present, the mechanical properties of most 3D-FDM printed polymeric parts often do not meet the requirements of industrial applications [13].

Hence, this paper aimed at evaluating the stiffness of produced lattice structures under compression, both numerically and experimentally, to make a comparison between the consistency assumed by finite element software and layering effects of experimental samples.

## 2. Fused Deposition Modeling on Lattice Structures

### 2.1. Design

Lattice structures were made by means of tessellation of a unit cell repeating it along the three main directions, essentially creating a honeycomb structure; see Figure 1. As known, honeycomb structures offer great advantages, especially in terms of minimum weight and great resistance to high stresses [14].

**Figure 2:**
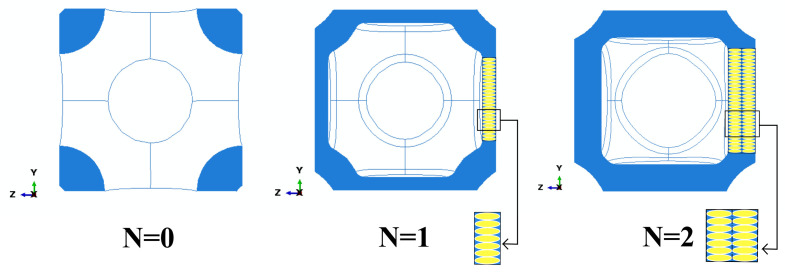
Section of unit cells: wall’s contours.

Three configurations of lattice structures were designed and analyzed, starting from three different unit cells with the same relative density, respectively, open cell, closed thin-walled cell, and closed thick-walled cell. These configurations of cells were designed based on the design for additive manufacturing. In detail, in accordance with the printing parameters used in the laboratory shown in Table 1, in particular the minimum thickness, the unit cells were produced, as illustrated in Figure 2.

It is immediately evident that the number of adjacent contours, N, in the walls of cells varies from 0 for open cell, is equal to 1 for thin-walled type, and is equal to 2 for thick-walled type. This choice of wall sizes will be the determining feature of the best geometry in terms of specific stiffness since layer thickness is one of the various parameters influencing the 3D-FDM printed objects [15,16].

**Figure 3:**
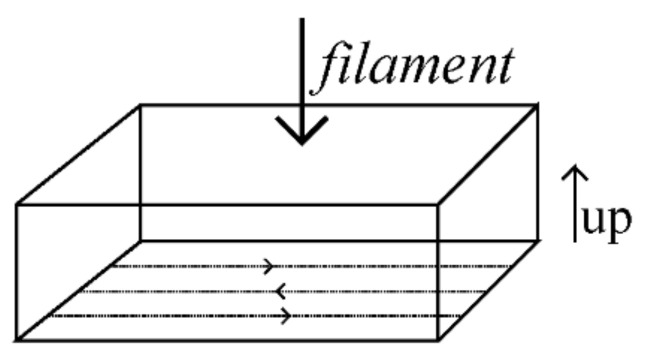
Building orientation of lattice structures.

The building orientation for all the configurations of lattice structures was the horizontal building direction, shown in Figure 3. This is another important printing process parameter in the mechanical response of the printed samples since it is found that building orientation plays an important role in the compressive strength of FDM printed parts [17,18].

**Figure 4:**
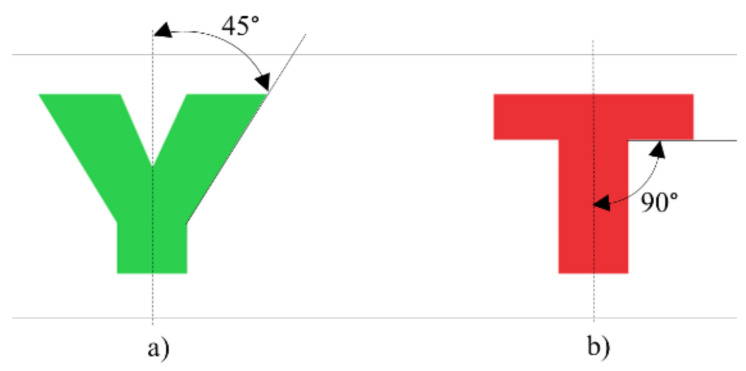
Overhang angle: (**a**) does not need support structures; (**b**) needs support structure.

The greatest advantage of these designed lattice structures, in addition to lightness, is that no support structures are required during the printing phase. This is due to the maximum overhang angle of 50 degrees of the used MEX 3D printer machine (Figure 4) (High speed 3D printing research center, National Taiwan University of Science and Technology, Taipei, Taiwan). In fact, 3D printers use a barely appreciable horizontal offset between consecutive layers. In this way, the upper layer does not perfectly overlap the underlying layer but stacks with this small offset, allowing to print overhangs with respect to the vertical of an overhang angle that varies from machine to machine, in a range from 20 to 70 degrees. In any 3D printer, the test for identifying the maximum overhang angle that allows printing without support is called the “massive overhang test”.

Hence, the 3D printing of self-supported structures is definitely convenient for saving material, printing time, and post-printing processing. There is also no risk of damaging the printed object when removing the support structure.

### 2.2. Experimental Tests

Three specimens of each configuration of lattice structure were printed by MEX 3D printer Flashforge dreamer© with TPU 90A filament. Monotonic compression tests were then performed on lattice structures under displacement control of 5 mm/min for three deformation levels, respectively, 10, 20, and 30% of the specimens’ height. For each configuration, load-displacement data were plotted in one graph to understand the geometry effect on compression behavior, in particular the number of contours with specific stiffness.

**Figure 5:**
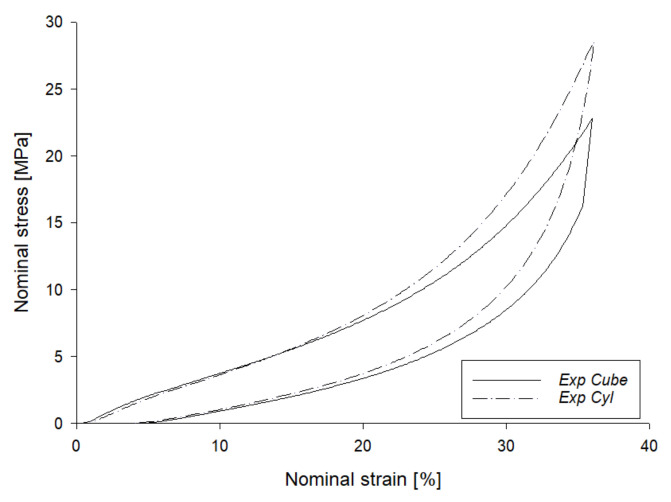
Compression-release tests on cubic and cylindrical TPU FDM samples.

This was performed while bearing in mind that geometry in FDM parts is an important factor that influences the stress distribution and mechanical properties of samples. Preliminary tests were carried out on cubic and cylindrical TPU 90A FDM samples for different deformation levels at the same strain rate. In order to highlight differences in the mechanical behavior of the two geometric configurations, the nominal stress-strain curves corresponding to 36% of deformation, shown in Figure 5, were obtained using the classical Equations (1) and (2) as follows:

(1) $$\sigma =\frac{L}{A_{0}}$$

(2) $$\varepsilon =\frac{d}{h_{0}}$$

where L is the compression applied load, $A_{0}$ is the cross-sectional area of the samples, d  is the displacement, and $h_{0}$ is the initial height of the samples.

The geometric configuration’s significant effect on the mechanical behavior of FDM printed parts became suddenly clear. In particular, at the beginning of the test, the cubic sample shows a higher Young’s modulus with a consequently lower plateau regime than the cylindric one. Experimental tests were performed by a servo-hydraulic MTS 810 with a 100 kN load cell. The load was applied perpendicularly to the building orientation of lattice structures so that the layers tend to crush each other, not stressing the weaker adhesion layer. In fact, in FDM printing, an anisotropic layering effect occurs, i.e., the printed parts are stronger in one direction than in the other [19]. 

A compression test is useful for unveiling the isotropic or anisotropic behavior of printed objects [20]. Given its layer-by-layer nature, the FDM process and many other 3D printing technologies, by definition, give rise to anisotropic parts in their structure [21,22]. This is due to the printing technique: when placing one layer on top of another, the underlying layer begins to partially solidify, causing incorrect and incomplete adhesion between the layers. This tends to create voids between the two adjacent layers, giving the printed object a consequent stiffness depending on the load application direction and also leads to its anisotropic behavior [23]. Obviously, this is one of the main difficulties in using and studying objects obtained by additive processes. Furthermore, the anisotropy present in FDM printed parts have different properties depending on the process parameters used in the printing phase [24]. This can have major consequences on the mechanical and functional properties of the printed parts in industrial applications.

### 2.3. FDM Process in Conjunction with TPU

As previously mentioned, the FDM additive process does not ensure mechanical properties equal to those of traditionally manufactured objects. This is certainly due to the considerable variability of the numerous process parameters of this type of 3D printing. Each of these parameters, as well as their combination, has different impacts on the mechanical properties of printed parts [25,26,27]. Through an extensive literature review, many FDM process parameters, such as layer thickness, air gap, bed temperature, raster orientation, model temperature, building orientation, etc. [28], have been found to influence the compressive strength of the samples obtained with the same technology. It must be noted that in all these works, the material used for printing is mostly ABS, while TPU has never been studied. This is of interest in our research, as TPU is increasingly used in conjunction with 3D printing in the manufacturing of thermoplastic printed parts since it offers a wide range of applications. This is due to the capability of TPU to combine mechanical performance characteristics of rubber with the possibility of being processed as a thermoplastic material.

At the same time, TPU is also a very complex material due to its hyperelastic and viscoelastic properties, hygroscopic nature of its filament, and wide range of values that its Young’s modulus can assume (from 10 to 2000 MPa).

## 3. Finite Element Method on Lattice Structures

### 3.1. Material: Models

**Figure 6:**
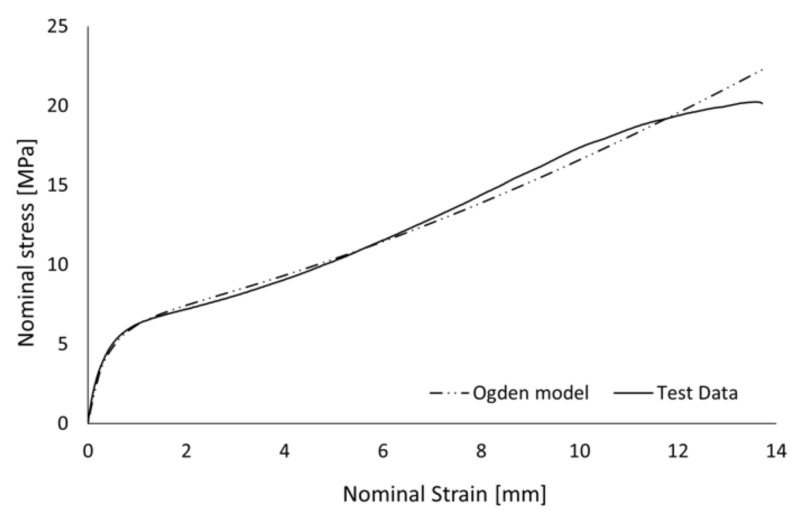
The test data for the second-order Ogden model vs. dog bone sample.

To simulate the behavior of lattice structures in TPU, an advanced model of hyperelastic material with hysteretic capability was defined in the Abaqus/CAE software (2020, Simulia Dassault Systèmes, Vélizy-Villacoublay, France). Uniaxial tensile test data from a TPU 90A dog bone sample were included in the Abaqus material model to identify the best match between experimental behavior and the different strain energy potential models available in the software, in the strain range of interest. It was found that in the strain energy potential model, described by means of Equation (3), the best approximate value of the experimental nominal stress-strain trend is the second-order Ogden model (Figure 6).

(3) $$U^{def}=\overset{N}{\underset{i=1}{\sum }}\frac{2\mu_{i}}{\alpha_{i}^{2}}\left({\bar{\lambda }}_{1}^{\alpha_{i}}+{\bar{\lambda }}_{2}^{\alpha_{i}}+{\bar{\lambda }}_{3}^{\alpha_{i}}-3\right)+\overset{N}{\underset{i=1}{\sum }}\frac{1}{D_{i}}{\left(J_{el}-1\right)}^{2i}$$

**Figure 7:**
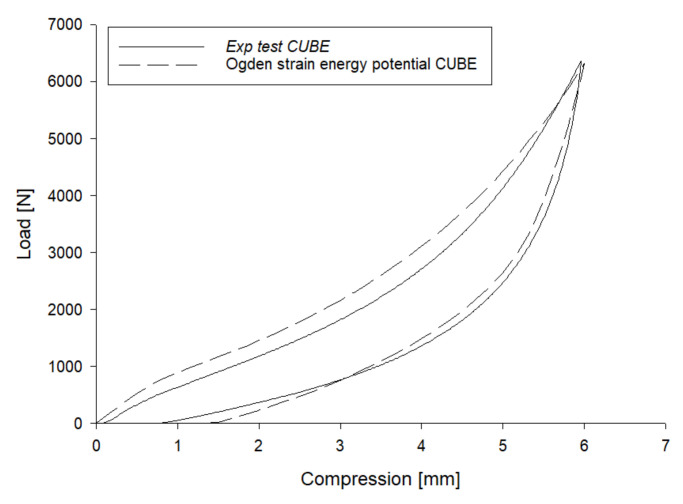
Determination of the hysteresis cycle.

The strain energy potential $U^{def}$ of Ogden form is expressed through the parameters $J_{el}$, ${\bar{\lambda }}_{i}$, $\mu_{i}$, $D_{i}$, $\alpha_{i}$ which represent, respectively, the elastic volume ratio, the deviatoric principal stretches, and the temperature-dependent material parameters, illustrated in Table 2. Using the second-order Ogden model and a compression-release test performed on a cubic FDM TPU sample, the best hysteresis loop was reproduced by modifying the Abaqus hysteresis parameters until the loop that best approximates the real behavior was reached (Figure 7). Numerically, in fact, the hysteretic behavior is governed by the following formulation:

(4) $${\dot{\epsilon }}_{B}^{cr}=A{\left[\lambda_{B}^{cr}-1+E\right]}^{C}{\left(\sigma_{B}\right)}^{m}$$

where the effective creep strain rate, and also the mechanical response, is described by means of two networks, A and B—network A identifies the equilibrium-relaxation part and network B, the non-linear one. In particular, for the term definitions of network B, $\lambda_{B}^{cr}-1$ represents the nominal creep strain, and *σ_B_* is the effective stress. Table 3 shows the editable parameters in the Abaqus formulation for Equation (4), identified in the literature [29,30], and after an optimization procedure on cubic and cylindrical numerical models, *S* is the stress scaling factor, m is an exponent usually bigger than 1, C is an exponent that can assume values from −1 to 0, and A and E are constants.

Voids left in the structure by additive technique and layering effects also need to be considered and analyzed in depth in FE analysis. FDM, as previously mentioned, generates an anisotropic, layered structure. This was considered by defining a local orientation of the lattice structure. Material structure orientation was used in conjunction with a new material model, i.e., an anisotropic elastic material model with an “engineering constants” option, to compare the isotropic and anisotropic behaviors.

By means of the second-order Ogden model, the isotropic behavior was analyzed since the FE software considers structures as isotropic solid. Simulations were also performed for a linear elastic material, both for anisotropic and isotropic materials, to show the possible effects of anisotropy on the mechanical response of the samples.

A linear elastic anisotropic material model was then adopted, in order to tune and reproduce the experimental results. The engineering constants that define this model—namely, elastic modulus, Poisson ratio, and shear modulus, in three principal directions (Table 4) were arbitrarily chosen on the basis of literature data [31,32], imposing a level of anisotropy of 50%. Here, the direction of the applied load was set as the second direction, while the first and third directions were the axes that defined the layer plane. In this way, the intra-filament elasticity was halved, simulating the FDM layer deposition effect.

### 3.2. Simulations and Overviews

**Figure 8:**
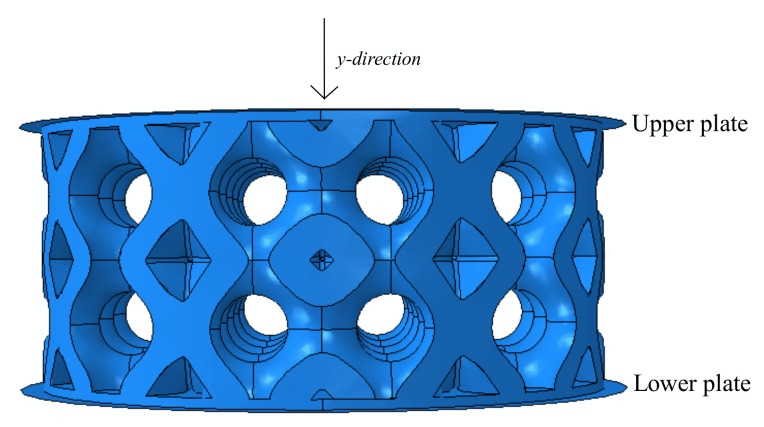
Analytical rigid plates and preferential direction y.

To simulate these tests in the Abaqus/CAE environment, the lattice structures were placed between two rigid analytical plates. The load was applied to the reference point of the upper plate by means of an imposed displacement (Figure 8). Three deformation levels were simulated for each geometrical configuration, which were, respectively, 10, 20, and 30% of specimens’ height, at the same strain rate of 5 mm/min.

**Figure 9:**
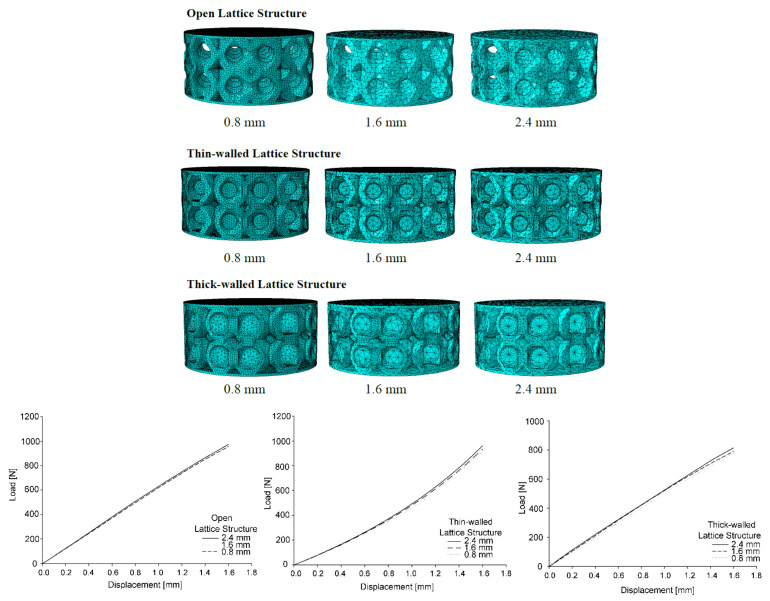
Mesh independency analysis of lattice structures and convergence analysis.

After a variability analysis that did not show major effects in a range from 0.025 to 0.3, a friction coefficient equal to f=0.1 was defined between the contact surfaces. Finally, the lattice structure’s typologies were formed as meshed units with linear solid tetrahedral elements, using a hybrid formulation that governs incompressible behaviors. The average mesh size of lattice structure elements was chosen as 1.6 mm, to have a good compromise between computational times and accuracy of the mechanical response in the simulations. This was carried out after a mesh independency analysis on varying the average mesh size from 0.8 mm to 2.4 mm, which showed no noticeably major changes in the mechanical response of the structures (Figure 9). Table 5 shows the resulting numbers of nodes and elements for the three geometrical configurations of lattice structures.

Load-displacement curves were obtained, as the reference point is affected by the sum of every single nodal response in the contact surface. From these curves, stiffness was calculated as the slope of the line that best fits the loading curve [33].

## 4. Results

**Figure 10:**
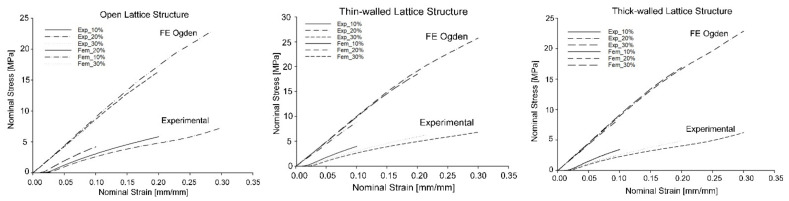
Nominal stress-strain curves of lattice structures, experimental and numerical.

All the obtained results, both experimental and numerical, are reported in Table 6. The nominal stress-strain curves in the range of interest, shown in Figure 10, were plotted through Equations (1) and (2), where the cross-sectional area of each typology of structures, $A_{0,\;eq}$, was calculated as follows:

(5) $$A_{0,\mathrm{eq}}=\frac{V_{L}}{h_{0}}$$

where $V_{L}$ is the volume of lattice, and $h_{0}$ is the height of the specimen.

In accordance with the results shown in Table 6, the comparison between experimental and numerical nominal stress-strain curves highlighted a good reproduction of the real experimental trend by Ogden model simulations, even if a visible gap was apparent between them.

**Figure 11:**
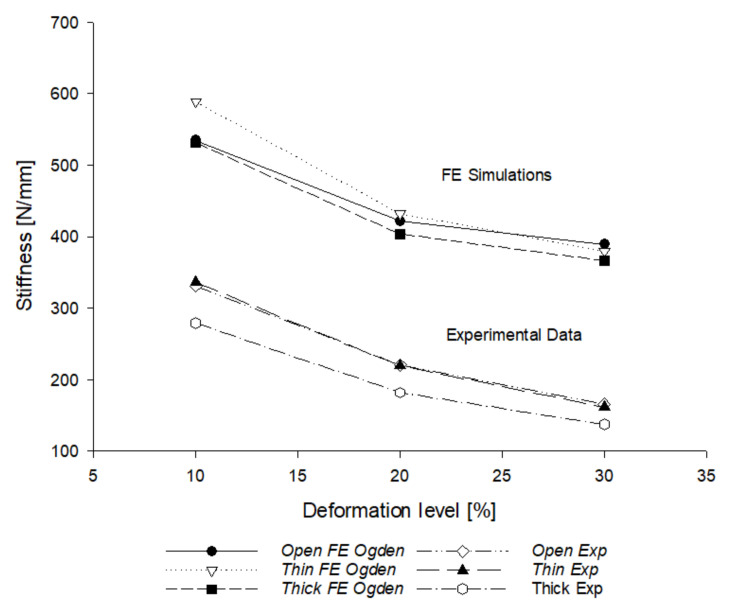
FEM Ogden model vs. experimental results.

**Figure 12:**
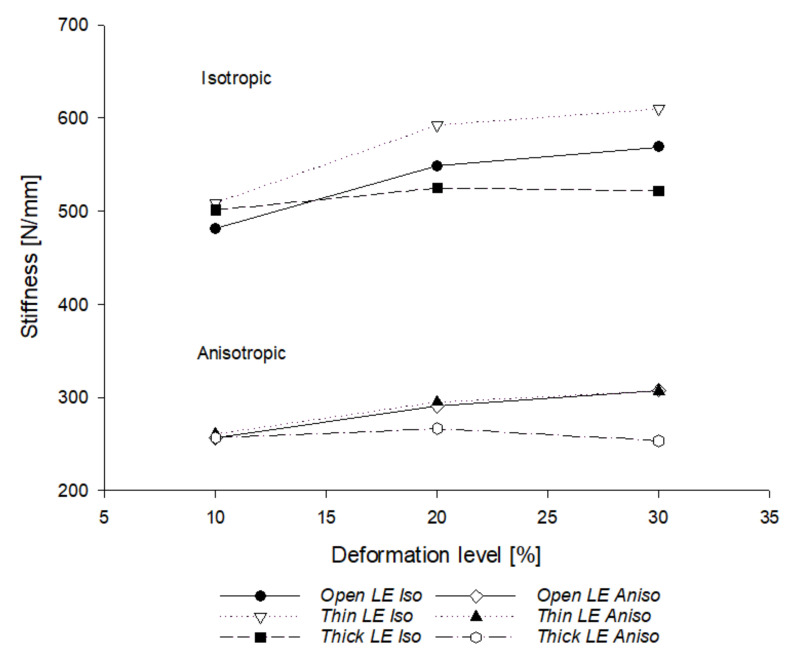
FEM simulations with linear elastic material: isotropic vs. anisotropic.

By plotting the stiffness results obtained for the hyperelastic model and experimental tests in a distinctive graph (Figure 11), it was revealed that the Ogden model is able to reproduce the exact trend of the experimental response of the lattice structures, but with a discrete gap. The same gap was also found between the isotropic and anisotropic linear elastic results (Figure 12). The linear elastic material model proved useful for identifying and studying the effects of 3D printing on the mechanical response of FDM printed parts.

The two graphs in Figure 11 and Figure 12 are similar; in fact, the simulation of the structures as isotropic cell solids showed a similar stiffness trend, but in the numerical results, it was double, compared to the anisotropic one. Therefore, the discrepancies between the experimental and numerical (by Ogden formulation) results are attributable to an anisotropic factor due to the printing process, i.e., to the various effects that occur in the printing phase. On the other hand, the simulations reflected the different mechanical responses of the three different geometrical configurations. For this reason, the aforementioned anisotropic layering factor is studied in depth in this discussion.

Geometrically, the graph in Figure 11 is able to highlight the lattice structure with the best mechanical response. In fact, in terms of stiffness, the thin-walled closed lattice structure showed the best performance, followed by the open typology, which showed a very similar mechanical response, especially in experimental tests. Finally, the thick-walled lattice structures showed a performance level always lower than the other two types, both numerically and experimentally.

Discrepancies in geometrical configurations response are certainly due to the effects of the FDM process [34]. In fact, as evident in Figure 2, the thick-walled structure had more deposited material in the walls, so it was more likely to have a high percentage of voids and layering effects inside. For this reason, it was desirable that these structures were weaker than the others, due to the greater and consistent presence of defects inside them.

## 5. Discussion

To understand the causes of discrepancies between experimental and numerical tests, all phenomena occurring in the printing process that influence the mechanical property of the FDM printed lattice structures were studied in depth. Several previous papers have found how FDM additive manufacturing technology widely affects the mechanical properties of printed parts [35,36]. FDM process is almost demanding due to the variability of its parameters and the uncontrollable printing effects such as porosity and layering.

Firstly, the porosity was studied both with SEM analysis and weight analysis. In their work, Abbot et al found that the simulated printed parts were at least 50% more solid than experimental printed samples [37]. This statement is in line with what was found in the present work, precisely shown in Figure 11, but it was found that this outcome is not due to the porosity of the FDM printed parts but to the layering effect that knocks down the mechanical properties of the additively manufacturing samples. In fact, SEM analysis on lattice structures conducted by Kumar et al. [38] revealed how the voids left by the FDM process have no major effects on the mechanical and functional properties of these structures. Moreover, through a weight analysis between real and virtual samples, no considerable differences in weight were found.

Finally, in the printing phase, there is another effect that is generated in the printed parts—the layering effect. It is extremely complex to simulate this phenomenon through a numerical model. For this reason, an *anisotropic layering factor* was identified, $\varphi_{l}$, which is able to describe how this effect acts on stiffness. Considering the similar precautions in the pre-printing phase (for example, care for the hygroscopicity of the TPU) and the same printing conditions for all the samples (i.e., the same process parameters on a unique batch and controllable boundary conditions), the focus was on the post-printing effects. In this way, all printed lattice structures were produced at the same time; hence, they had the same aging time, always showing comparable characteristics. Following a phenomenological approach, by defining $\varphi_{l}$ as a function of geometry (i.e., number of wall’s contours, *N*) and imposed deformation  ε, Equation (6) can be written as

(6) $$\varphi_{l}=\alpha \left(N\right)\cdot e^{\beta \cdot \varepsilon }$$

where $\alpha \left(N\right)$ is a function of the number of contours *N* in a cell’s wall, equal to $\alpha =1.3+\left(0.15N\right)$, β is a constant equal to 0.02, and ε is the strain level in percentage (i.e., 10, 20 and 30%).

Firstly, the real trend of experimental curves was reproduced by means of a regression model with the R-squared coefficient of 0.98. Consequently, the parameters of Equation (6), α and β, were identified through an iterative process on varying the critical parameters that adversely affect stiffness, N and ε. Finally, when the optimal match between curves was reached, $\varphi_{l}$ was described as used in Equation (6).

**Figure 13:**
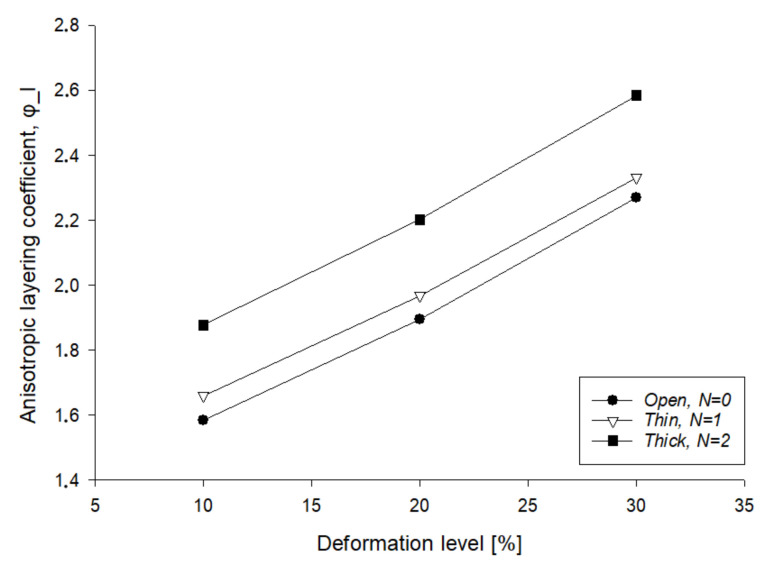
Anisotropic layering factor trend for each configuration.

The plot of Equation (6) reveals an increasing effect of the number of layers *N* on the deformation level (Figure 13). This is due to the fact that with the increase of deformation, the stresses acting on layers and printing defects increase, determining a drastic decrease in the stiffness.

**Figure 14:**
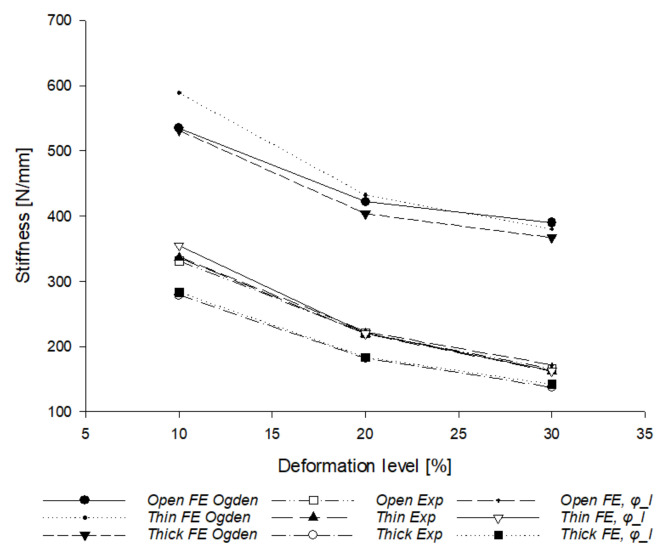
Experimental results and FE simulation results with the Ogden model.

**Figure 15:**
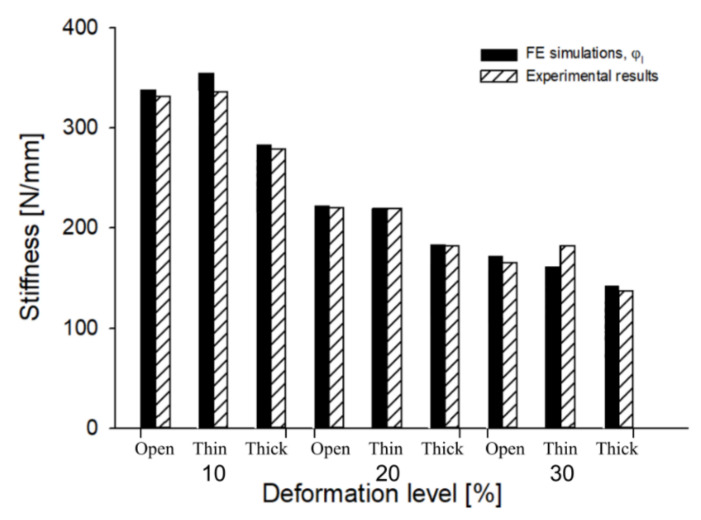
Experimental results and FE simulations corrected with $\varphi_{l}$.

Now, after recalibration of the simulated curves obtained by the Ogden hyperelastic model by dividing the stiffness values by the $\varphi_{l}$ factor, a very good agreement can be found with the experimental results, as presented in the plot of Figure 14 and more precisely in the bar plot of Figure 15.

## 6. Conclusions

This study was conducted in order to analyze and understand the mechanical behavior of specimens of cellular structures obtained by additive deposition process fused in thermoplastic polyurethane. Particular attention was paid to the layer-by-layer deposition effect on the resulting stiffness, showing that the mechanical behavior of printed samples with this technology is not easily predictable. Major findings can be summarized as follows:

- By the traditional FE analysis, an anisotropic behavior of such structures was proven;
- Anisotropy was ascribed to the layering process of filament, not always quantifiable a priori;
- A phenomenological layering factor *φ_l_* was defined that tries to correlate the number of FDM contours, the deformation level, with the anisotropy degree;
- On the basis of the layering factor, thin-walled cell structures were confirmed to be the less affected, whereas larger walled structures were negatively affected;
- The mechanical and functional behaviors of this kind of structure were confirmed to be influenced by many parameters, related to material and process, as well asa specific geometry.

## Figures and Tables

**Table 1:** Printing process parameters.

Printing Phase: FDM Parameters	
Nozzle diameter [mm]	0.4
Layer height [mm]	0.2
Printing speed [mm/min]	1100
Print infill [%]	100
Printing temperature [°C]	230
Bed temperature [°C]	70
**FDM Machine Parameters**	
Minimum thickness [mm]	0.6
Maximum overhang angle [°]	50

**Table 2:** The parameters of the second-order Ogden model.

Material Parameter	$\mu_{i}$	$D_{i}$	$\alpha_{i}$
i = 2, order	6.1298	0.0000	−1.9004

**Table 3:** Abaqus hysteresis parameters.

Parameter	S	m	C	A	E
value	2.2	4	0	12 × 10^−3^	0.01

**Table 4:** Engineering constants: Young’s modulus, Poisson ratio, and shear modulus in the three principal directions.

Material	$E_{1*}$	$E_{2*}$	$E_{3*}$	$v_{12}$	$v_{13}$	$v_{23}$	$G_{12}$	$G_{13}$	$G_{23}$
Linear elastic	13	26	13	0.49	0.39	0.49	4.36	9.35	4.36

* 1: x direction; 2: y direction; 3: z direction.

**Table 5:** Mesh of lattice structures: nodes and elements.

Topology	Nodes	Elements
Open cell	24,779	104,274
Closed thin-walled cell	33,735	145,385
Closed thick-walled cell	37,790	157,045

**Table 6:** Specific stiffness of lattice structures.

10%	Stiffness K_0_ [N/mm]			
	EXP	FEM		
		Ogden	Isotropic LE	Anisotropic LE
Open cell	331	534.6	480.8	256.4
Closed thin walled	336	588.8	508.7	260.7
Closed thick walled	278.9	531.2	500.8	256.3
**20%**	**Stiffness K_0_ [N/mm]**			
	EXP	FEM		
		Ogden	Isotropic LE	Anisotropic LE
Open	220.3	421.7	548.1	290.2
Closed thin walled	219.2	432.2	592	294.8
Closed thick walled	181.9	403.8	524.6	266.2
**30%**	**Stiffness K_0_ [N/mm]**			
	EXP	FEM		
		Ogden	Isotropic LE	Anisotropic LE
Open	165.5	389.6	568.4	307.7
Closed thin walled	161.7	380.1	609.5	306.6
Closed thick walled	137.5	366.7	521.6	253.3

## Data Availability

Data are available on request due to restrictions eg privacy or ethical.
